# Oral manifestations of anaplastic large cell lymphoma: clinicopathological features and main immunophenotypic findings of 3 cases

**DOI:** 10.1007/s12308-026-00703-8

**Published:** 2026-05-29

**Authors:** Carla Isabelly Rodrigues-Fernandes, Isabela Assis de Siqueira, Cinthia Veronica Bardález López de Cáceres, Juan Manuel Arteaga Legarrea, Victor Tieghi Neto, Geovana Martins Lopes, Marcelo Cavalcanti da Cruz, Hélder Antônio Rebelo Pontes, Wilson Delgado-Azañero, Guilherme Rossi Assis de Mendonça, Pablo Agustin Vargas, Fábio Luiz Coracin, Felipe Paiva Fonseca

**Affiliations:** 1 Oral Pathology Unit, Department of Clinic and Preventive Dentistry, Universidade Federal de Pernambuco, Recife, Pernambuco Brazil; 2 Department of Dentistry, Department of Pathology, Department of Hematology - Barretos Cancer Hospital, Barretos, São Paulo Brazil; 3 Department of Oral Surgery and Pathology, School of Dentistry, Universidade Federal de Minas Gerais, Belo Horizonte, Brazil; 4 Service of Oral Pathology, João de Barros Barreto University Hospital, Federal University of Pará, Belém, Brazil; 5 Departamento de Patología, Medicina y Cirugía Oral, Facultad de Estomatología, Universidad Peruana Cayetano Heredia, Lima, Perú; 6 Department of Pathology, School of Medical Sciences, University of Campinas, Campinas, Brazil; 7 Oral Diagnosis Department, Piracicaba Dental School, University of Campinas, Piracicaba, Brazil

**Keywords:** Non-Hodgkin lymphoma, Anaplastic cell lymphoma, Anaplastic lymphoma kinase, Mouth

## Abstract

Anaplastic large cell lymphoma (ALCL) is contained within a larger category of aggressive mature T-cell lymphomas. Their subclassification relies on the presence or absence of *Anaplastic Lymphoma Kinase* (ALK) gene fusions and other molecular alterations. Cases in the head and neck and mouth are uncommon. The aim of this manuscript is to describe the clinicopathological and immunohistochemical features of a series of anaplastic large cell lymphoma (ALCL) affecting the oral cavity. Three cases are included in this series, all of them affecting male patients, with a mean age of 27.7 years old. Concomitant involvement of the oral cavity and lymph nodes was known to occur in two cases, while cutaneous lesions were not recorded in any patient. The neoplasms more often presented as solitary or multiple painful swellings in the gingiva. All cases were positive for CD30 expression, and the two cases investigated for CD3 were negative. ALK protein was expressed in two cases, and it was negative in one case. EBER was negative in the two cases investigated. One patient was known to decease despite chemotherapy. In conclusion, oral manifestations of ALCL are very rare and more commonly represent a disseminated disease.

## Introduction

Anaplastic large cell lymphoma (ALCL) encompasses different subtypes with distinct clinical and genetic features [1], comprising a group of aggressive mature T-cell non-Hodgkin lymphomas (NHL). It is composed of pleomorphic, anaplastic cells, with a cohesive growth pattern, tendency to invade lymph node sinuses, and strong CD30 expression, which was originally described by Stein et al. in 1985 [2] and initially termed as Ki-1 lymphoma [3–5].

The latest World Health Organization classification of hematolymphoid tumors (WHO-HAEM5) recognizes different variants of ALCL. According to the presence of *Anaplastic Lymphoma Kinase* (ALK) gene fusions, the tumors are subclassified into ALK-positive ALCL and ALK-negative ALCL [3, 4, 6]. Breast implant-associated ALCL (BIA-ALCL) is now recognized as an independent disorder and no longer considered a provisional entity [6, 7]. The International Consensus Classification (ICC) also recognizes *DUSP22*-rearranged ALK-negative ALCL as a genetic subtype of systemic ALK-negative ALCL [7]. Otherwise, the cutaneous counterpart of ALCL is classified within the spectrum of primary cutaneous CD30-positive T-cell lymphoproliferative disorders, which present clinicopathologic and prognostic features distinct from systemic ALCL [6].

ALCL is relatively uncommon, comprising approximately 12% of all peripheral T-cell lymphomas (PTCL) [8]. Advances in the investigation regarding the pathobiology of ALCL have progressed, providing new information about clinical, microscopic, immunophenotypic, and genetic features [9, 10]. Clinically, ALK-negative ALCL predominantly presents as a nodal disease, whereas ALK-positive ALCL affects extranodal sites in up to 60% of patients [1]. Primary cutaneous ALCL represents a well-defined entity characterized by solitary or localized lesions and a 10-year disease-specific survival rate of approximately 90% [6].

It has been suggested that CD30-positive T-cell proliferative disorders involving mucosal sites share similarities with their cutaneous counterparts, considering histopathology and phenotype, raising the possibility that they represent analogous manifestations within the same biologic spectrum [11]. However, unlike primary cutaneous ALCL, oral cavity involvement represents an important diagnostic challenge, particularly in distinguishing primary mucosal disease from systemic ALCL with secondary oral involvement, which has significant therapeutic and prognostic implications.

In the oral cavity, very few cases of ALCL have been described to date [12], which makes important to further document and better define the spectrum of oral cavity ALCL, its association to both systemic and cutaneous CD30-positive T-cell lymphoproliferative disorders. Therefore, the aim of the current report is to describe an additional series of three ALCL affecting the oral cavity and to provide an updated literature review on this clinical presentation.

## Clinical history

**Fig. 1:**
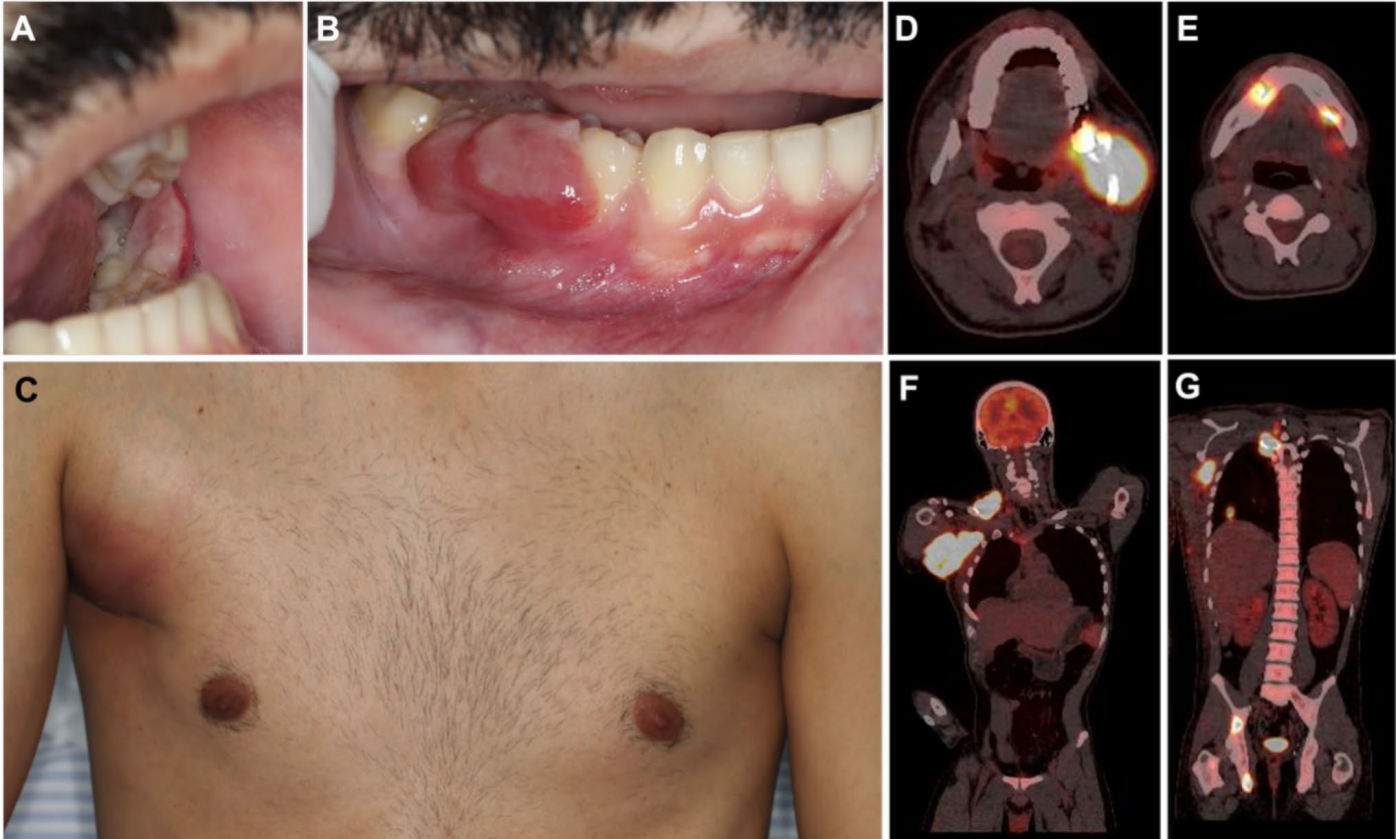
Clinical features and PET-CT findings of ALK-positive ALCL (Case no. 3). **A** 24-year-old male presenting two ulcerated swellings with irregular surface involving the lower gingiva. One lesion is located in the left posterior gingival mucosa and the other **B** affected the right gingival mucosa, covering both vestibular and occlusal faces of the right pre-molars and the first molar. **C** The same patient presented a reddish to purplish swelling in the right axillary region with hard consistency and fixed implantation. **D** Tumor hypermetabolism captured in the left masticator space and **E** and in the mandible. **F** Several areas of hypermetabolism involving cervical and axillary lymph nodes, right chest wall, lungs, **G** right ischiopubic branch, and skeleton

Detailed information regarding demographic and clinical features of each patient is available in Table 1. All cases affected males, with a mean age of 27.7 years old (range 11–48 years). Two cases were known to involve the oral cavity and lymph nodes simultaneously. Two cases demonstrated intraoral ulcerated nodules (cases no. 1 and 3). Extraoral examination of these patients also revealed facial asymmetry (case no. 1) and an axillary swelling (case no. 3) (Fig. 1). Associated symptoms included trismus, toothache, shoulder pain, jaw pain, and B-symptoms. Patients reported rapidly growing tumors, ranging from 1 to 11 months. Computed tomography (CT) of patient no.1 demonstrated involvement of cervical, pre-auricular and occipital lymph nodes. Positron emission tomography-computed tomography scan (PET-CT) of patient no. 3 showed hypermetabolism areas in multiple lymph nodes, left cervical masticator space, lungs, chest wall, T3 vertebra, and right ischiopubic branch. Information regarding treatment and follow-up was only available for one patient (case no. 3), who was submitted to chemotherapy with CHOP regimen (cyclophosphamide, doxorubicin hydrochloride, vincristine, and prednisone). After 7 months of follow-up, this patient died due to sepsis.

**Table 1:** Clinicopathological features and main immunohistochemical findings and EBV status of the three cases reported in this study

Case	Age/sex	Site of involvement	Signs	Symptoms	B-symptoms	Duration (months)	IHC/EBER ISH results	Final diagnosis
1	48/M	Buccal vestibule/upper gingiva/lymph nodes	Swelling/ulcer	Trismus/pain	No	1	CD3−, CD20−, CD30 +, CD45RO +, Granzyme B +, Perforin +, Ki67 (55%), EBER−, ALK-	ALK-negative, ALCL
2	11/M	Maxilla	NA	NA	NA	11	CD3−, CD20-, CD30 +, CD43 +, Granzyme B +, Perforin +, EMA +, Ki67 (85%), EBER−, ALK +	ALK-positive, ALCL
3	24/M	Gingiva/lymph nodes/other organs*	Swelling/ ulcer	Toothache, jaw pain, shoulder pain	Yes	4	CD2 +, CD7 +, CD20−, CD25 +, PAX5-, CD30 +, EMA−, EBER−, ALK +	ALK-positive, ALCL

*M* male, *F* female, *NOS* not otherwise specified, *NA* not available

*Cervical masticator space, lungs, right chest wall. T3 vertebra, right ischiopubic branch; + positive,—negative

## Materials and methods

Stains were performed for CD20, CD3, CD30, CD45RO, cytotoxic marker (granzyme B and/or perforin), EMA and ALK. Other antibodies were also variably tested. Detailed information about the antibodies and the reactions are available in Table 1. The proliferative index of the tumors was determined by calculating the percentage of malignant cells with nuclear staining for Ki67 among 1000 tumor cells using a high-power view in five hot-spot fields. In situ hybridization (EBER-ISH) was performed to search for the presence of EBV.

## Results

**Fig. 2:**
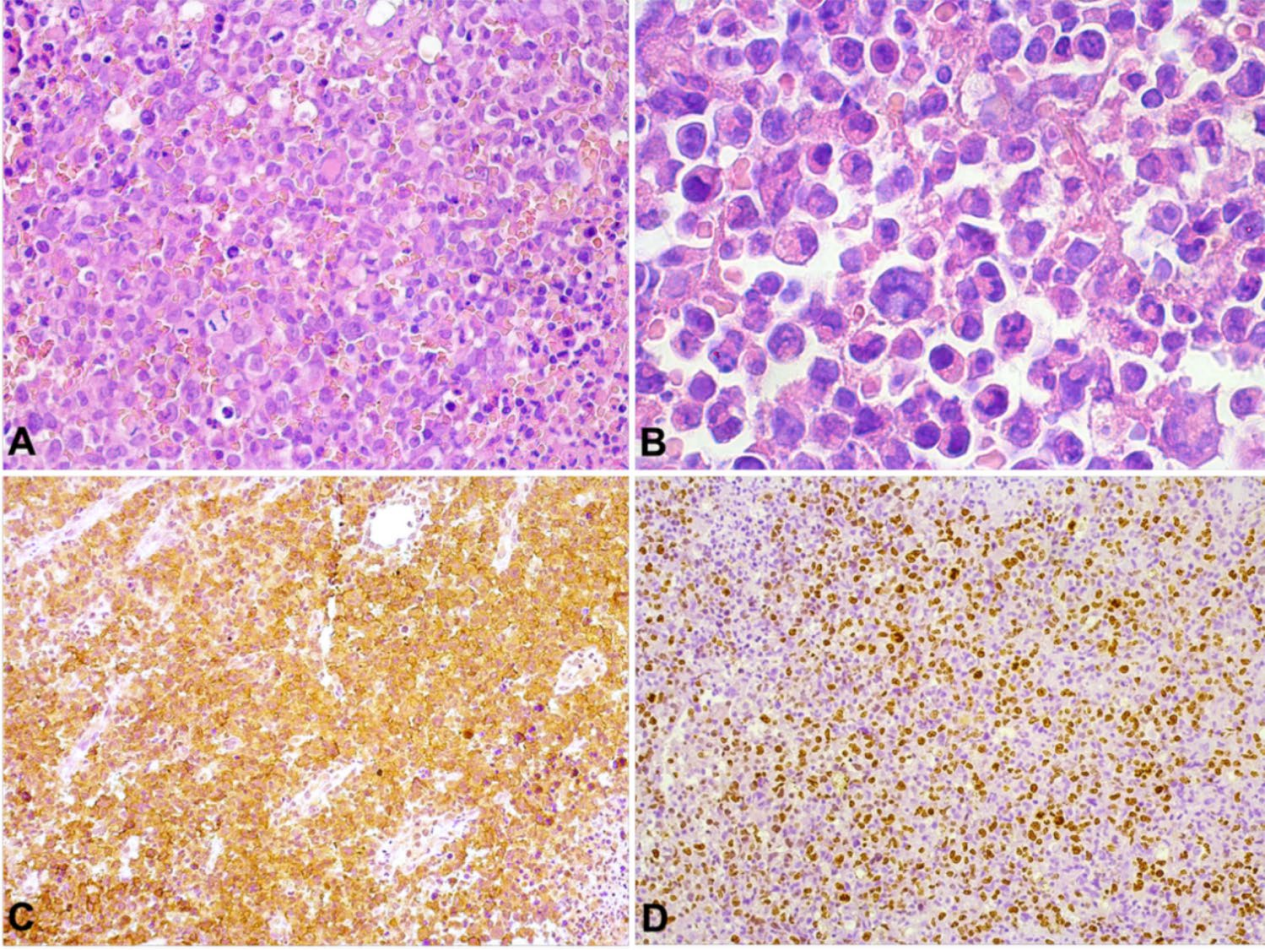
Microscopic and immunohistochemical features of ALK-negative ALCL. **A** Similar to the observed for ALK-positive ALCL, the neoplasm exhibits a diffuse proliferation of tumor cells with a high degree of pleomorphism, including the presence of hallmark cells (H&E, 200x). **B** High-power view of the tumor showing hallmark cells alongside with large pleomorphic cells demonstrating large and eccentric nuclei and multinucleated cells (H&E, 400x).** C** Positive membranous and Golgi-like expression of CD30 in the neoplastic cells (DAB, 200x). **D** High proliferative index demonstrated by nuclear Ki67 staining (DAB, 200x)

**Fig. 3:**
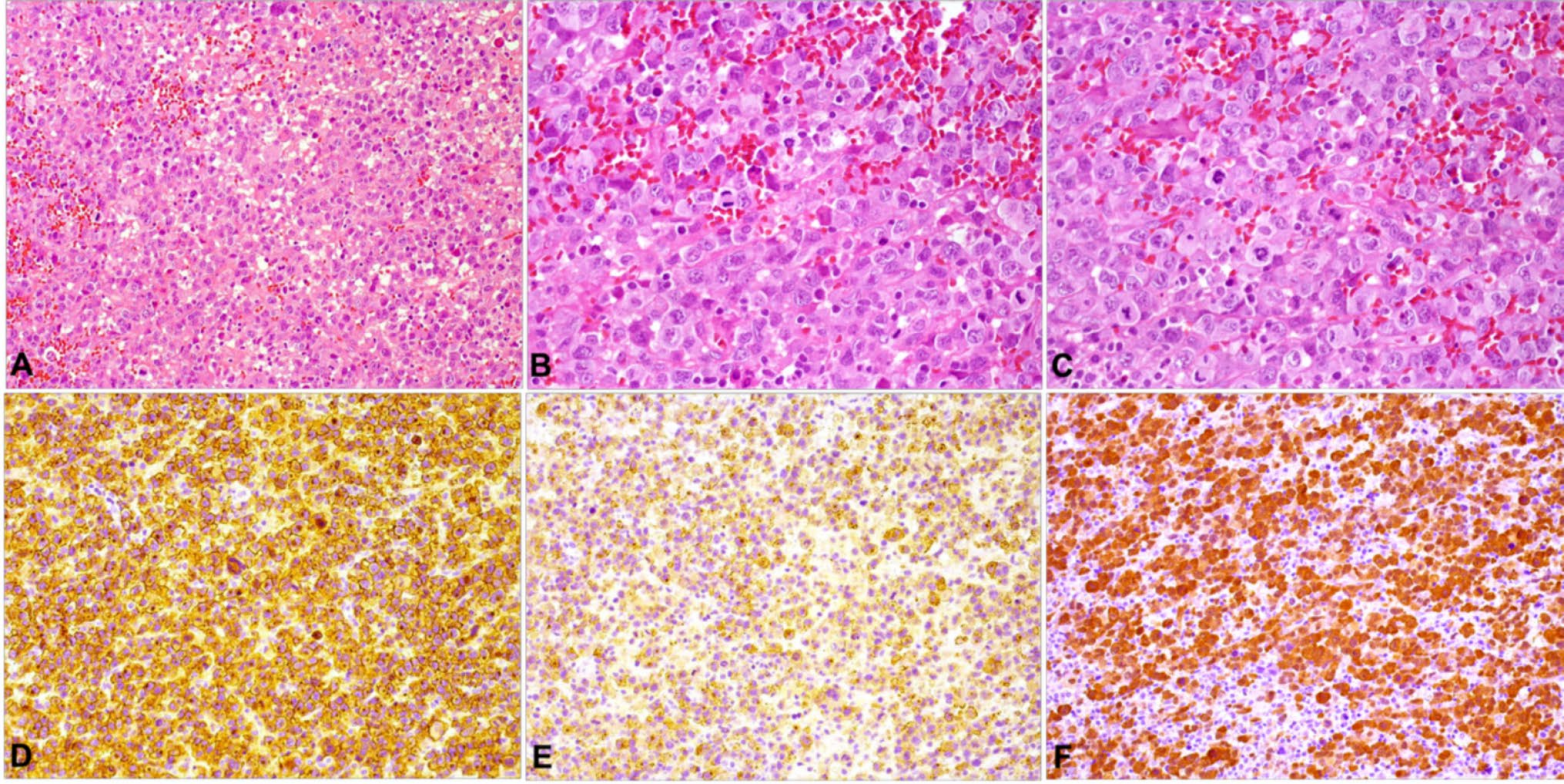
Microscopic and immunohistochemical features of ALK-positive ALCL. **A** Diffuse proliferation of neoplastic lymphoid cells exhibiting marked cytoplasmatic and nuclear pleomorphism **(**H&E, 200x).** B** Neoplastic cells exhibiting vesicular nuclei and multiple nucleoli. There are also cells with eccentric kidney-shaped nuclei (hallmark cells) and abundant eosinophilic cytoplasm (H&E, 400x). **C** Frequent atypical mitotic figures are also visualized (H&E, 400x).** D** Diffuse cytoplasmic expression with Golgi-accentuation of CD30 in neoplastic cells (DAB, 200x). **E** Positive expression of EMA in large neoplastic cells (DAB, 200x). **F** Nuclear and cytoplasmatic staining of ALK protein in tumor cells (DAB, 200x)

Microscopic findings are illustrated in Figs. 2 and 3. Tumors exhibited a diffuse proliferation of medium to large pleomorphic cells with clear and eosinophilic abundant cytoplasm. The cells displayed vesicular and irregular nuclei with numerous, small nucleoli. The largest cells either presented with eccentric kidney/horseshoe-shaped nuclei (hallmark cells) or multiple nuclei. “Doughnut cells” were also present in all cases. Atypical mitotic figures were frequent, and an angiocentric arrangement of neoplastic cells was observed in two cases.

The reactions confirmed the T-cell origin of the tumors by the variable expression of CD45RO, granzyme B, perforin, and/or CD2 and CD7 proteins while the two cases investigated for CD3 resulted negative. All cases showed positivity for CD30 with the typical strong membranous and Golgi-accentuated pattern. Two cases were diffusely positive for EMA and one resulted negative. Considering the expression of ALK, two cases were classified as ALK + ALCL and one case was diagnosed as ALK-negative ALCL. The mean proliferative index of the tumors demonstrated by Ki67 ranged from 55 to 85%. EBER ISH was negative in all cases.

## Discussion

Clinicopathological information concerning oral manifestations of ALCL is currently very limited [11, 12], and the report of new cases will contribute to better understand this uncommon clinical presentation.

ALCL are uncommon, more often diagnosed in North America and Europe [13–17]. There are currently no well-established etiologic agents and despite some cases reported in HIV + patients, ALCL is not considered an HIV-related lymphoma [1, 18]. ALK-positive ALCL is defined by the expression of ALK protein which results from chromosomal translocations in the ALK gene, more often the *NPM::ALK* rearrangement [19]. Meanwhile, most cases of ALK-negative ALCL have been shown to demonstrate mutations in *TP53, TP63, JAK/STAT3* and *DUSP22* genes, although the occurrence of these molecular abnormalities has not yet been used to define a new variant of ALCL by the WHO, while the ICC group recognizes *DUSP22*-R (locus on 6p25.3) ALK-negative ALCL as a genetic subtype of systemic ALK-negative ALCL, which has been described in 30% of the cases [20, 21]. In other cases of ALK-negative ALCL, recurrent rearrangements in *TP63* locus on 3q28 encode p63 fusion proteins, most commonly with TBL 1XR1, as a result of inv(3)(q26q28); they have been observed in 8% of the cases [8, 22, 23].

While ALK-positive ALCL is more prevalent among children and young adults, the median age at presentation of ALK-negative ALCL is 54 years. In our series, ALK-positive cases affected patients with 11 and 24 years old, while the ALK-negative case affected an older individual (48 years-old). ALCL exhibits a male predominance, as corroborated in our series [14, 24]. As we observed, most patients are diagnosed in advanced stages [25]. Extranodal involvement is also common and is more frequently observed in the skin (primary cutaneous ALCL should be ruled out), soft tissues, bone, lungs, liver, bone marrow and central nervous system [1, 25]. Cases in the head and neck seem to be unusual; a study with 1,306 patients with primary extranodal ALK-positive ALCL showed that only 134 tumors (10.3%) affected this region [25]. Head and neck cases include the nasopharynx [26], scalp [27], and external auditory canal [17]. A study on mucosal CD30-positive T-cell disorders described 15 cases affecting the oral mucosa, orbit, conjunctiva, and paranasal sinuses, with or without involvement of other sites. Of these, one patient had tongue and skin lesions, although it was not clear what specifically affected the tongue, since primary cutaneous ALCL is mainly a skin-limited disease [11]. In accordance with this series, oral ALCL has been diagnosed mostly in the gingiva and tongue, clinically manifesting as rapid-growing, ulcerated swellings, associated or not with pain. Our literature review showed that most of the reports also described the involvement of other sites in addition to the oral cavity manifestation; however, in most cases this information is not available [28–30] (Table 2).

**Table 2:** Demographic and clinicopathological features of oral ALCL cases published in the literature

Clinicopathological variables	*n* = 31	%
Sex		
Female	9	29.0
Male	21	67.8
ND	1	3.2
Age (mean age: 50 yrs)		
< 50 yrs	15	48.4
> 50 yrs	15	48.4
ND	1	3.2
Oral site		
Gingiva	11	35.4
Tongue	5	16.0
Palate	4	12.9
Lip	5	16.1
Other^1^	5	16.1
ND	1	3.2
Involvement of other sites		
Yes	10	32.2
No	5	16.1
ND	16	51.6
Symptoms		
Pain	6	19.3
Asymptomatic	2	6.5
Other^2^	1	3.2
ND	22	70.1
Clinical presentation		
Swelling	6	19.3
Ulcer	4	12.9
Swelling and ulcer	9	29.0
ND	12	38.7
Final diagnosis		
ALCL	11	35.5
ALK-negative, ALCL	17	54.8
ALK-positive, ALCL	3	9.7
Treatment		
Chemotherapy	16	51.6
Radiotherapy	4	12.9
No treatment	4	12.9
Other^3^	1	19.3
ND	6	19.3
Status		
Alive	21	67.7
Dead	2	6.5
ND	8	25.8

^1^Maxilla (1); Mandible (1); Alveolar ridge (1); Gingiva + mandible (2); Gingiva + maxilla (2)

^2^Pruritus

^3^Chemoterapy + bone marrow transplant

In addition to the presence of neoplastic cells with an eccentric, kidney/horseshoe-shaped nuclei, ALK-positive ALCL tumors may exhibit other patterns, including small-cell, lymphohistiocytic, composite, and Hodgkin-like forms [3]. Regarding differential diagnosis, the small-cell pattern can be misdiagnosed as peripheral T-cell lymphoma not otherwise specified (PTCL NOS), and the Hodgkin-like pattern resembles nodular sclerosing Hodgkin’s lymphoma [24, 31]. It is also important to document EBV negativity, excluding the differential diagnosis of extranodal T/NK lymphoma [4]. ALK-negative ALCL carrying *DUSP22* rearrangement more often displays a sheet-like growth pattern, less pleomorphic cells and frequent doughnut cells [10].

ALCL is consistently positive for CD30 expression that is expressed in the majority of tumor cells [8]. Most of the cases aberrantly lack mature T cell markers like CD2, CD3, and CD7, while CD43 and CD45RO are commonly positive. Cytotoxic markers like TIA1, granzyme B, and perforin are usually positive, even in the absence of CD8 expression, but are frequently absent in *DUSP22* rearranged ALK-negative ALCL cases. EMA and MUM1 proteins are observed in most of the cases. Proliferative index is usually high [24, 31]. ALK expression is unexpected in primary cutaneous ALCL and is more commonly associated with the systemic disease [6]. This marker may be observed in the cytoplasm, nucleus, and nucleolus depending on the molecular translocation present in each case [9, 31, 32].

A study which performed and immunohistochemistry and fluorescence in situ hybridization on ALCLs found a 5-year overall survival rate of 17% in cases with *TP63* rearrangement in comparison to ALK-positive ALCL (95%) [23]. Investigation of *STAT3* found similar results, regardless of ALK status, while ALK-negative ALCL cases harboring *STAT3* and/or *JAK1* mutations presented a reduced overall survival [33]. Gene rearrangement may also influence the morphologic aspects of ALCL; modifications on *JAK2* are associated with the presence of large anaplastic cells [6, 8]. Simultaneous rearrangements of *TP63* and *DUSP22* in ALCL showed primary refractory disease and death due to disease progression [21]. Pedersen et al. found that 62 cases of ALK-negative ALCL with *DUSP22* rearrangements presented a 5-year overall survival of 80%, which are more similar to ALK-positive ALCL mean survival rate (85%) [22]. On the other hand, another study with 12 samples showed a 5-year overall survival of 44% [24]. Unfortunately, it was not possible to investigate the molecular basis of our three cases, although this analysis could help us to better appreciate the molecular signature of these neoplasms. In addition, only one case of our series had available information regarding treatment and outcome, which impaired further comparisons with other studies involving mucosal and/or mucocutaneous CD30-positive T-cell lymphoproliferative disorders.

Like other T-cell lymphomas, treatment for ALCL usually consists of CHOP or CHOP-like regimens [1, 25, 29]. This therapy seems to provide a better prognosis for ALK-positive ALCL than to its negative counterpart, despite the outcome of our patient [32]. Other agents are emerging, including velemetostat and idelalisib; for cases of ALK-positive ALCL, a selective ALK inhibitor named crizotinib has demonstrated a good response in in patients with relapsed/refractory disease [4, 34]. Nevertheless, these possibilities remain far from clinical context.

Oral manifestations of ALCL are very rare and affected patients should be investigated for the presence of a disseminated disease. Moreover, a possible primary cutaneous or breast implant-associated ALCL, as well as other peripheral T-cell lymphomas, should also be ruled out.

## Data Availability

No datasets were generated or analysed during the current study.
